# Unraveling lipid and inflammation interplay in cancer, aging and infection for novel theranostic approaches

**DOI:** 10.3389/fimmu.2024.1320779

**Published:** 2024-02-01

**Authors:** Daniel Conde-Torres, Alexandre Blanco-González, Alejandro Seco-González, Fabián Suárez-Lestón, Alfonso Cabezón, Paula Antelo-Riveiro, Ángel Piñeiro, Rebeca García-Fandiño

**Affiliations:** ^1^ Departamento de Física Aplicada, Facultade de Física, Universidade de Santiago de Compostela, Santiago de Compostela, Spain; ^2^ Organic Chemistry Department, Centro Singular de Investigación en Química Biolóxica e Materiais Moleculares (CiQUS), Universidade de Santiago de Compostela, Santiago de Compostela, Spain; ^3^ MD.USE Innovations S.L., Edificio Emprendia, Santiago de Compostela, Spain

**Keywords:** cancer, infection, aging, lipidomics, chronic inflammation

## Abstract

The synergistic relationships between Cancer, Aging, and Infection, here referred to as the CAIn Triangle, are significant determinants in numerous health maladies and mortality rates. The CAIn-related pathologies exhibit close correlations with each other and share two common underlying factors: persistent inflammation and anomalous lipid concentration profiles in the membranes of affected cells. This study provides a comprehensive evaluation of the most pertinent interconnections within the CAIn Triangle, in addition to examining the relationship between chronic inflammation and specific lipidic compositions in cellular membranes. To tackle the CAIn-associated diseases, a suite of complementary strategies aimed at diagnosis, prevention, and treatment is proffered. Our holistic approach is expected to augment the understanding of the fundamental mechanisms underlying these diseases and highlight the potential of shared features to facilitate the development of novel theranostic strategies.

## Graphical Abstract

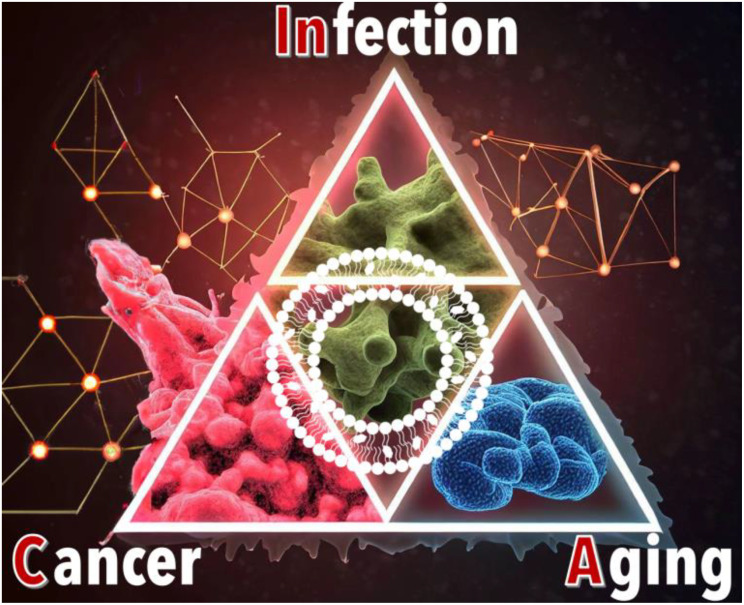

## 1 Introduction

Cancer-Aging-Infection (CAIn) represent the three vertexes of a triangle that is responsible of most of the disease-related deaths in the modern world (1). It is alarmingly disappointing that while over 20 million publications on cancer-related topics exist, failure rates of about 90% are revealed for the treatment of solid tumors (2). The global spread of antibiotic resistance and the terrifying consequences left by viral agents such as SARS-CoV-2 also indicate that much remains to be done in the field of infection (3). On the other hand, the price that must be paid for survival is getting old, with the surcharge that it entails in the form of pathologies associated with aging and degeneration (4).

Although cancer, aging and infection undoubtedly go hand in hand, the global cross-link between these pathologies is unclear. They have traditionally been treated as independent entities, making our knowledge about them fragmentary, chaotic and confusing. There is a vast quantity of published data on CAIn diseases separately, and a growing number of studies pointing out the correlations between the three vertices of this triangle (5). However, the available information is ineffectively integrated, making it difficult for researchers to draw conclusions able to develop effective prevention programs and treatments. Therefore, there is an urgent need for a systematic understanding of CAIn and its linkages—as well as their similarities and differences—to develop new strategies that improve public health.

**Figure 1:**
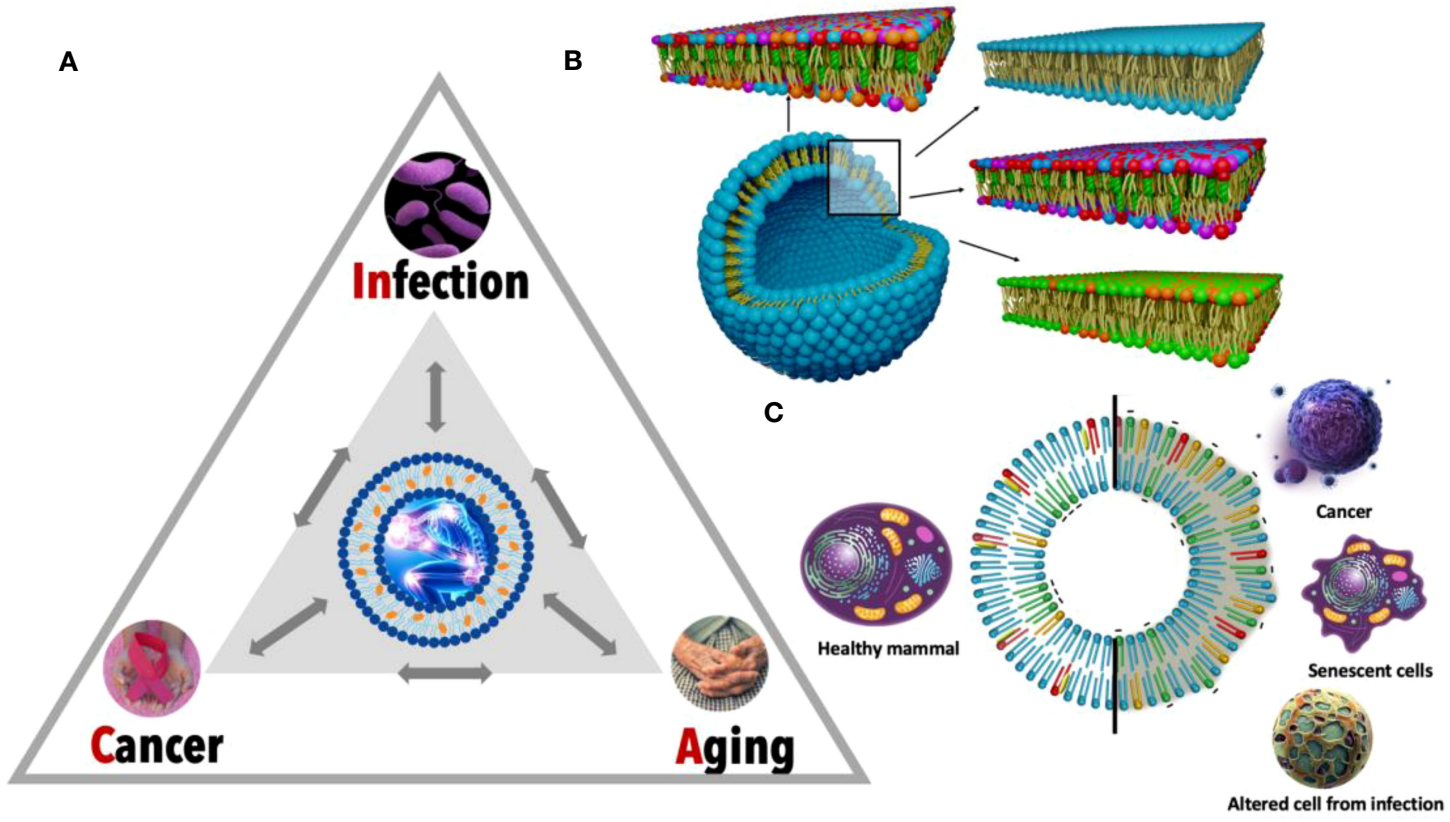
Detailed view of the CAIn triangle and specific lipid membrane alterations. **(A)** The CAIn Triangle model is depicted, illustrating the interconnectedness between infection, cancer, and aging. Each point of the triangle signifies a condition, with the directional arrows indicating the complex and mutual interactions, which underscores the possibility of each condition influencing the onset of the others. **(B)** This panel features a detailed cross-section of lipid bilayers representing a segment of the cellular membrane structure. The diverse colors correspond to different lipid molecules, showcasing the intricate and organized arrangement within a typical cellular membrane. This organization is disrupted in pathological conditions, which is captured by the altered patterns in the lipid bilayer, signifying inflammation and its role in cellular dysfunction. **(C)** The circular graph contrasts the lipid composition of healthy cell membranes (on the left) with those exhibiting lipid dysregulation in CAIn-affected cells (on the right). This visual dichotomy illustrates the lipidomic variations that correlate with the transition from normal cellular function to pathological states, providing a molecular perspective on the role of lipid changes in aging and disease.

Upon examining the CAIn Triangle, one of the most conspicuous connections that emerges between its three vertices is chronic inflammation (**Figure 1A**). This omnipresent factor not only characterizes cancer, but also serves as a significant risk element for aging and age-associated diseases (6, 7). Continuous exposure to infection leads to inflammation, contributing to the development of cancer (8, 9), and the connection between inflammation and infection is further supported by research literature highlighting the upregulation of endogenous retroviruses (HERVs) in inflammatory diseases (10, 11). Despite the clear connection of chronic inflammation, there’s another common denominator between the three vertices of the CAIn Triangle which has been largely overlooked: the lipidic alteration of the cellular membranes of the involved cells (**Figures 1B, C**). The recent explosion of lipidomics unveiled unique lipid profile singularities in cancer (12), bacterial and viral infections (13), as well as senescent cells (14), underlining the necessity for more in-depth exploration. Consequently, modifications in the lipid composition of affected cells are proposed as a relevant biomarker for monitoring, with potential applications in prevention, diagnosis, and treatment.

In this work, we seek to explore and decipher the complex relationships within the CAIn Triangle, ultimately outlining a comprehensive strategy for the prevention, diagnosis, and treatment of these related pathologies. Emphasizing the importance of viewing the CAIn Triangle as an interconnected system, we advocate for more efficient research collaborations. Such an approach is expected to lead to the development of groundbreaking prevention programs and therapeutic interventions. This holistic perspective not only aims to revolutionize the study of these individual conditions, but also to reshape strategies for addressing broader public health issues and disease management. By doing so, this comprehensive approach has the potential to transform our combat against these diseases, making a lasting impact on global health.

## 2 Tracing the links: exploring individual connections in the CAIn Triangle

### 2.1 Connections between cancer and infection

**Figure 2:**
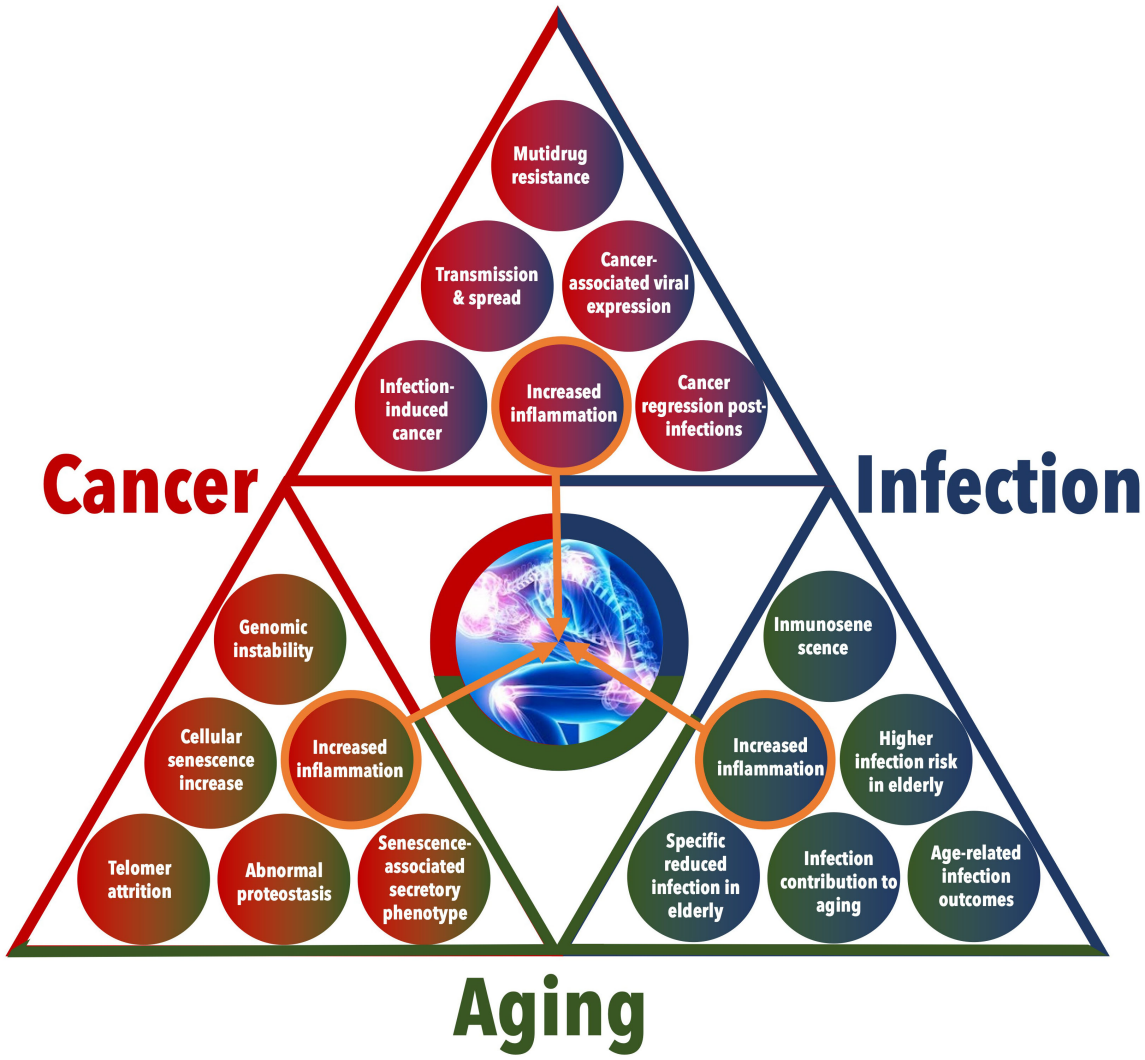
Detailed interactions among Cancer, Infection, and Aging. This figure provides an in-depth visualization of the multifaceted relationships and feedback loops between cancer, infection, and aging. The triangular layout emphasizes the interconnected nature of these conditions, with each side of the triangle representing the interaction between two conditions and with a central focus on increased inflammation as a critical link among the three.

The relationship between cancer and infection is complex and bidirectional (**Figure 2**). Certainly, one could view cancer as a chronic infectious disease – the result of an unresolved invasion and proliferation of a “foreign” entity within the body. This perspective is reinforced by the relevance of numerous concepts applicable to the treatment of both chronic infections and tumors, such as multi-drug resistance (15). The striking resemblance between chronic infections and cancers is further emphasized by instances where cancers are, in fact, infectious agents. Notable examples include the transmissible tumors found in Tasmanian devils and bivalves (16) as well as the transfer of certain cancer types to transplant recipients (17).

Epidemiological studies have yielded several indications of a connection between cancer and infection. They indicate that infections and inflammatory responses are connected to 18% of the global cancer burden, with estimates ranging from 7% in developed countries to about 22% in developing countries (18). Different infectious agents, such as viruses, bacteria, fungi, and parasites might trigger the development of cancer. *Helicobacter pylori* was found to be the most frequent infection in the world and the principal responsible for gastric cancer (19). These bacteria are able to adapt to the extremely acidic environment of the stomach, leading to infection and damaging the gastric mucosa, provoking gastric pathogenesis and molecular and phenotypic changes, causing cancer. Recent studies demonstrate the effectiveness of eradicating *Helicobacter pylori* for gastric oncogenesis prevention (20). Oncoviruses play a major role in both the initiation and progression of cancer, being responsible for nearly 12% of human cancers (21). Certain oncoviruses can integrate their DNA to the host genome, triggering the creation of oncoproteins and consequently provoking both the inactivation of tumor suppressor genes and the activation of oncogenes. For example, individuals with human immunodeficiency virus (HIV) infection are at an increased risk of developing immune deficiency syndrome (AIDS), as well as higher susceptibility to liver, lung, oropharyngeal, and anal cancers, along with Non-Hodgkin’s lymphoma (NHL) or Hodgkin’s lymphoma, compared to the general healthy population (21). Concretely, Kaposi sarcoma (KS) is the most frequent neoplasm among HIV patients, usually related to immune suppression. HIV is considered a crucial cofactor in KS pathogenesis, as it has been demonstrated that the employment of antiretroviral therapy reduces KS incidence (22).

On the other hand, cancer patients present a higher vulnerability to acquire infectious diseases than the general population (23). The human organism´s ecosystem, microbiota, and the host, usually establish a symbiotic relationship, performing vital functions such as pathogens defense or nutrients production. Since the immune system is meant to maintain a balance with the microbiota and to fight against most microbial invasions, immunocompromised patients are at an increased risk of infection (24). Immunosuppression may be induced by cancer itself or by some therapies, such as chemotherapy. Chemotherapy, besides being one of the most effective treatments for metastatic cancers, modifies the interactions between the host and the microbiota, increasing the susceptibility of infection, and therefore being considered an immunosuppressive treatment (21). In that manner, therapy may increase the risk of carcinogenesis since an altered microbiota can lead to gene overexpression and therefore may trigger complex diseases, including cancer. Due to this fact, certain infections in cancer patients compromise their correct response to therapy. Neutropenic fever is an important cause of morbidity and mortality during chemotherapy of acute myeloid leukemia (25), while acute urinary tract infections are common within prostate cancer patients (26). Patients treated with chemotherapy have also an increased risk of acquiring invasive fungal infections, most of them caused by *Candida* species and *Aspergillus fumigatus* (27). On the other hand, *C. albicans* infections increase the risk of carcinogenesis and metastasis (28).

In conditions involving altered epigenetic mechanisms, like cancer, numerous genes exhibit modified expression patterns, and previously suppressed genes become active. In healthy adult cells, human endogenous retrovirus type K (HERV-K) expression is typically inhibited. However, in tumor cells, there is a notable increase in HERV-K mRNA expression (29, 30). The upregulation of HERV-K expression in tumor cells can lead to the activation of previously inhibited genes. This, in turn, can result in the production of viral proteins that contribute to tumor formation and growth. Additionally, the presence of HERV-K-derived antigens may stimulate immune responses that target tumor cells, further highlighting the complex interplay between cancer and infection.

Considering the significant conceptual parallels between infections and cancer, it’s thought-provoking to examine how research in infectious diseases has influenced cancer immunotherapy strategies since William Coley’s pioneering work in the late 19th century. He revealed that high fevers induced by erysipelas infections could lead to remission in certain tumors (15). In the landscape of immunotherapy, Bacillus Calmette–Guérin (BCG) represents a significant milestone (31). Originally developed as a vaccine against tuberculosis, BCG is an attenuated strain from *Mycobacterium bovis*. Its role in cancer treatment became evident when it was shown to be the first effective immunotherapy against established human bladder cancer, offering substantial improvement over other treatments for intermediate and non-muscle invasive bladder cancer. The success of BCG in immunotherapy is attributed to its ability to induce a long-lasting antitumor immune response, which has been a breakthrough in the field. Curiously, in a recent study exploring the intersection of COVID-19 and cancer, researchers found unexpected instances of temporary or prolonged clinical remission from cancer following SARS-CoV-2 infection, despite the generally severe outcomes of COVID-19 in cancer patients (32). Sixteen case reports were examined, most of which involved remission after viral infections, with a few cases following anti-SARS-CoV-2 vaccination. The immune response to COVID-19 was identified as a possible player in both tumor regression and progression, underscoring the need for further investigation into the potential benefits of oncolytic viruses and mRNA vaccines in treating tumors resistant to other forms of immunotherapy.

Hence, cancer and infection go hand by hand, and it should be possible to take advantage of their connection for both the prevention and treatment of cancer. Understanding these complex interactions is crucial for the development of novel cancer treatments, particularly in the realm of immunotherapy.

### 2.2 Connections between aging and infection

It is a widely known phenomenon that the elderly population has a high prevalence of infectious diseases compared to younger adults (33). As life expectancy increases globally, the fact that common diseases become risk factors for the population represents a major challenge to public health systems. This is the case of influenza (34), or the more recent COVID-19 pandemic, which has highlighted the vulnerability of the older segment of the population, both in terms of susceptibility to infection and outcome (35). Apart of COVID-19 and influenza, a range of infections is commonly encountered in the elderly population, including urinary tract infections, pneumonia, diverticulitis, endocarditis, bacteremia, nosocomial bacterial infections, and skin and soft tissue infections, notably diabetic foot infections (33). However, meningitis is infrequently observed among the elderly, with meningococcal and *Haemophilus influenzae* meningitis virtually non-existent. *Listeria monocytogenes* meningitis is rare but more common in this age group. Other less common infections in the elderly include tuberculosis, *Legionella pneumophila* and *Chlamydia pneumoniae* pneumonia, mycoplasma infection, and viral infections other than influenza, herpes zoster reactivation, and viral gastroenteritis (33). The microbial profile encountered during specific infections differs in the elderly population (33). These changes in microbiology may be attributed to age itself and/or underlying comorbidities. Notably, not only do the types of microorganisms differ during infections, but there is generally a greater diversity of pathogens in the elderly compared to younger adults.

The clinical presentation of common infectious diseases differs significantly between elderly and younger patients (33). In the elderly, infections tend to have fewer symptoms, and fever may be absent or reduced in severe cases. Non-specific manifestations such as falls, delirium, anorexia, or general weakness are common signs of infection in the elderly. However, these symptoms can also be present in non-infectious diseases, making it challenging to identify infectious diseases in this population.

The effect of aging on the immune system is known as *immunosenescence*. Immunosenescence is the progressive deterioration of the effectiveness of the immune system, affecting immune system organs, immune cells, and cytokines. This results in an increased prevalence of, among others, infectious diseases in the elderly population (36). The extent of changes in the immune system is a result of genetic, lifestyle factors, as well as of the presence of disease or of exposure to infectious agents (37). The most notable process that the immune system undergoes as the individual ages is the thymic involution, the loss of mass and compartmentalisation of the thymus, which is replaced by adipose tissue. This process starts at a very early age, 1-2 years, and undergoes a major decline that slows down in adolescence (38). The thymus is the main organ where lymphoid precursors mature into naive T cells, so that the age-related decline in thymus activity leads to a gradual decrease in the production of naive T cells (39). Naive T cells are those capable of recognising new pathogens, as opposed to memory T cells, which respond to antigens that the immune system has previously encountered. To compensate for the decline in thymic activity, T cell replenishment occurs through homeostatic renovation. This process is sufficient to maintain the population of naive CD4+ T cells –responsible for the activation and suppression of the immune response by the release of cytokines– but not naive CD8+ cells –cytotoxic cells, responsible of killing the infected cells– because of the different dynamics of activation, expansion, and apoptosis they undergo (40). In the balance of T cells in elders, memory T cells predominate due to the history of previous infections during their lifetime, especially those related to chronic infections such as *Cytomegalovirus* (41). Senescent memory T cells shows signs of cellular aging, such as short telomeres, lack of telomerase, and the expression of negative signalling receptors (42).

The increased prevalence of infectious diseases in the elderly population extends beyond the ineffectiveness of the immune system. The loss of capacity and functionality of some organs plays a key role in a normal prevention of the infection. The development of respiratory diseases is favoured by the joint effect of the weakening of the swallowing mechanism, the change of the bacterial flora in the oral cavity, or the use of drugs that alter the secretion of saliva, leading to an increase in the colonisation of infectious agents that then spreads to the lungs. The loss in lung elasticity and the decrease of the bronchiole diameter due to tissue alteration, together with a worse response of the respiratory system due to age-associated muscle weakening, hinder the elimination of harmful agents from inside the lungs (43), resulting in a higher prevalence of pulmonary infectious diseases in the elderly population.

The hormonal change brought about by the menopause is a contributing factor to the development of diseases, such as urinary tract infections. The population of *Lactobacilli*, the predominant microorganisms of the vaginal flora in premenopausal women that contribute to maintaining an acidic pH, is reduced after menopause. In their absence, the pH increases and, with it, the population of uropathogens, such as *Escherichia coli* or *Streptococcu* ***s***, associated with urinary tract infection increases (44). In men, the most prevalent cause of urinary tract infections is prostatic hypertrophy, where tissue growth causes obstruction of the urethra, hindering the flow of urine and thus favouring the presence of pathogens in the bladder (45). Common to both sexes is the development of urinary tract infections in patients with a chronic indwelling urethral catheter, favoured by the protection it offers against antimicrobial agents (46). All this exemplifies infections resulting from the treatment of age-related diseases and their complications. The prevalence of cardiovascular diseases also increases with age because of the declining health status of the body, favoured by bad health habits. Surgical implantation of pacemakers and implantable cardiac defibrillators is used to treat bradyarrhythmias and tachyarrhythmias. In addition to the risk of infection during the intervention, displacement of device components or discomfort to the patient may trigger infection, as well as reoperations for maintenance and replacement of the devices (47).

The relationship between aging and infection is also bidirectional (**Figure 2**) and many infectious diseases have been shown to increase the risk of developing age-related diseases and to contribute to the aging process (33). Such is the case of atherosclerosis, whose prevalence increases in patients who have suffered a previous infection with pathogens such as *Cytomegalovirus*, Herpes simplex virus (HSV), and *Chlamydia pneumoniae*; the development of Alzheimer’s following HSV and *C. pneumoniae infections*, or atrophic gastritis following *Helicobacter pylori* infection. Several possible models have been proposed to explain the relationship between aging and infection (33): direct tissue damage by pathogens, a trade-off between immune defense and tissue damage, and the contribution of latent or chronic infections to aging. Latent infections may periodically reactivate, leading to immune-mediated elimination of infected cells. In tissues with a low regenerative capacity, such as the brain, this process may result in significant cell loss over a lifetime. Microorganisms capable of causing chronic infections typically find ways to evade the immune response but may also contribute to the aging process through manipulation of cellular and tissue functions.

### 2.3 Connections between cancer and aging

At first glance, aging and cancer may seem like completely unrelated processes. The typical behavior of aged cells is characterized by low rates of proliferation and energy production, whereas cancer cells exhibit the opposite characteristics. However, molecular changes observed in aged cells can also drive and relate to tumorigenesis (48). More precisely, it has been suggested that senescence, a condition of permanent cell cycle stoppage linked to aging, is a form of antagonistic pleiotropy (49). This means that certain genes that are beneficial in early life can become harmful as one ages. Interestingly, there is a remarkable convergence between the hallmarks of cancer (50) and the hallmarks of aging (51) in numerous biological processes. These shared hallmarks encompass genomic instability, abnormal proteostasis, telomere attrition, heightened inflammation, and increases in cellular senescence, thus indicating the presence of intricate biological connections (**Figure 2**) (52).

With the progressive aging of society, the proportion of older individuals diagnosed with cancer is significantly rising. The majority of cancer cases occur after the age of 50 or 60, and individuals aged 70 and above bear approximately half of the global cancer mortality burden, resulting in around 5 million deaths annually (52). Several aging-associated factors could potentially explain this phenomenon. Firstly, the accumulation of oxidative stress and DNA damage over time, resulting from lifelong exposure to endogenous metabolic insults (such as free radicals) and exogenous factors (such as UV irradiation and certain foods), may contribute to cell transformation and tumor initiation (53). However, the link between aging and the likelihood of developing cancer is not only influenced by genetic changes, but also by epigenetics. Changes in epigenetics have been shown to increase the expression of oncogenes, silence tumor suppressors, and drive tumorigenesis (54). Many of the epigenetic alterations observed in age-related processes are also present in cancer, indicating a close relationship between the two (55, 56). For example, as age progresses, there is a general loss of histone proteins, chromatin remodeling, changes in histone modifications and changes in DNA methylation patterns (57). Multiple studies support the hypothesis that changes in histone expression can have an effect on the structure of chromatin, moving it away from the tightly packed heterochromatin form (57). It was demonstrated that changes happen in the occupancy of the H3 histone unit in mice as they grow older. These changes are associated with an increase in chromatin accessibility at proinflammatory genes (58). In a similar fashion, the balance of activating or repressive histone modifications has been shown to vary with age, and might be linked to life expectancy (59). The three-dimensional structure of chromatin is also subject to change during aging. It has been shown that, in human cells, chromatin is segregated into topologically associated domains (60), a characteristic that is lost in the Hutchinson-Gilford progeria syndrome, which is notable for causing premature aging (61). The most well-known epigenetic link between aging and cancer is the site-specific hypermethylation of developmental genes, which is a typical characteristic of aging tissues, and is also a key feature of multiple cancers (62). Recently, it has been found that the changes in DNA methylation in aged cells and cancer cells are more different than previously believed (63). Cancer cells tend to have bidirectional methylation, while aged tissues are more likely to have global methylation. In mammals, as organisms grow older there is a general decrease in DNA methylation across the genome, which leads to activation of normally silenced genes (64). Yet, it is still largely uncertain if these modifications affect genes associated with aging, or if the epigenetic shifts are the result of changes in these specific genes.

An additional factor implicated in both aging and cancer is the accumulation of senescent cells during the aging process. These senescent cells display a senescence-associated secretory phenotype (SASP) (65), which has been demonstrated to inhibit the growth of tumor cells through an inflammasome-mediated mechanism. This means that they release inflammatory mediators (such as interleukin (IL)-6, IL-8, monocyte chemoattractant protein (MCP)-2, growth-regulated oncogene alpha (GROα), etc.), potentially fostering a tumor-promoting environment. However, and counter-intuitively, the SASP can also promote tumor progression (45). Furthermore, malignant cells can affect normal cells in their vicinity, propagating a genomically unstable microenvironment to surrounding healthy tissues (66). During normal aging, there is a decline in immunity and the development of chronic non-severe inflammation (42), caused by the accumulation of both genetic and epigenetic changes throughout life. A clonal population of CD8+ T cells with an epigenetic signature developed in response to an aged host has been identified (67) and are thought to be able to promote neighboring cells to adopt a SASP. During aging, CD8+ memory T cells, a crucial subset for effective tumor clearance, undergo changes in chromatin structure which can lead to less accessible DNA. This can potentially result in impaired immune reaction due to the inaccessibility of regions important for the response of protein complex factors involved in DNA transcription and cell signaling (68). Also, regulatory T cells (Tregs) dysfunction can contribute too to immunosenescence through enhanced suppression of effector T cell response. A study of patients with head and neck squamous cell carcinoma found that older patients had increased Tregs in the peripheral blood but decreased tumor-infiltrating Tregs (69).

The interplay between aging and cancer is complex and multifaceted. While the accumulation of somatic mutations over time contributes to the increased risk of cancer initiation with age, it is not the sole explanation. Remarkably, certain long-lived species including certain bat species, elephants, and blue whales, exhibit remarkable cancer resistance, challenging the notion that aging inevitably leads to increased cancer incidence (70). These species have evolved various tumor-suppressing mechanisms that remain to be fully understood. Recent studies have shown that somatic mutation rates are slower in long-lived mammals, suggesting that evolutionary constraints may influence the accumulation of mutations (71). Therefore, the age dependency of cancer incidence is not solely driven by the passage of time but is also influenced by biological factors that are likely affected by the aging process. Moreover, as individuals age, the risk of cancer-related death rises until around age 90, after which it tends to stabilize or decrease. This could be due to the fact that people who live to be very old (over 100 years) usually have weaker inflammatory responses, which may protect them from certain age-related diseases with a strong connection to inflammation, including cancer (72–74).

## 3 The CAIn Triangle: chronic inflammation as a common denominator

**Figure 3:**
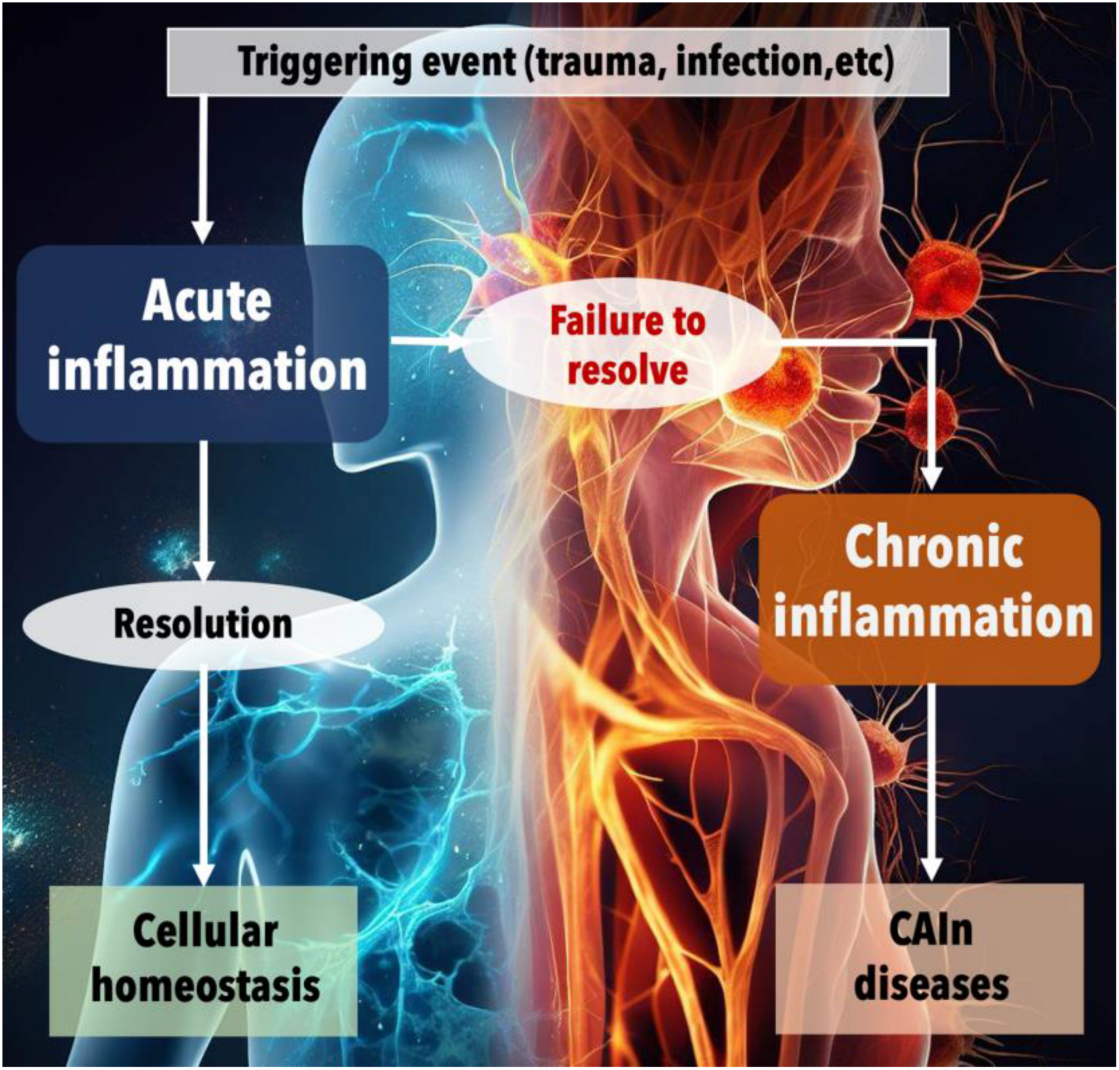
The progression from acute to chronic inflammation and the onset of CAIn diseases. This figure illustrates the physiological response to harmful events and the critical juncture between acute and chronic inflammation. The process begins with a triggering event, such as trauma or infection, which initiates acute inflammation—a normal, protective response aimed at healing the body. Under ideal circumstances, this acute phase is followed by a resolution, leading to the restoration of cellular homeostasis. However, when the inflammation fails to resolve, the condition can progress to chronic inflammation. This unresolved inflammation is depicted as a persistent, detrimental state that contributes to the development of various chronic diseases, collectively referred to as CAIn diseases, which include cancer, pathologies associated with aging, and the long-term consequences of infections.

Inflammation, a highly complex defense mechanism, is a key component of innate immunity present from birth in many organisms, including humans. It acts as a double-edged sword, offering both beneficial and detrimental impacts on the host. Ideally, in the context of acute infections, inflammation summons anti-microbial immune cells to the site of infection, leading to localized tissue destruction and transient systemic effects like fever. It initiates tissue repair and healing responses, eliminating infections or tumors and restoring homeostasis. However, the delicate equilibrium that exists between pro-inflammatory and anti-inflammatory responses can be disrupted, profoundly impacting disease progression and an organism’s healing capacity. When inflammation fails to resolve adequately or is improperly regulated, it ushers in a state of chronic inflammation (**Figure 3**). This condition, characterized by persistent, low-grade inflammation, is associated with an increased risk of certain diseases (75) and forms a pivotal link connecting chronic infections, cancer, and aging—vertices of the CAIN Triangle (**Figures 1**–**3**).

Mechanical or chemical trauma triggers acute infection, which, if resolved, restores cellular balance. Persistent inflammation leads to chronic inflammation and the development of chronic diseases like cancer, aging, and long-term effects of infections.

### 3.1 Chronic inflammation in infection

Inflammation can be triggered by several events, such as infections, breakdown of body tissue from traumatic events (like surgery or accidents), and conditions like autoimmunity or allergies (76). For example, the human body’s immediate response to an invasion of foreign pathogens, such as bacteria, viruses, or other microorganisms, is to initiate an inflammatory reaction. Essential to the body’s defense mechanism, this response involves activating and mobilizing immune cells to the infection site to neutralize and eliminate the invaders. In an acute infection scenario, inflammation serves as a key agent in the body’s defense, triggering a series of events, including vasodilation, increased blood flow, and the release of pro-inflammatory cytokines, all working collectively to isolate and eradicate the pathogen. Yet, there are situations where the body fails to eliminate the agent, causing the promotion of a state of low-grade, non-infective (that is, ‘sterile’) systemic chronic inflammation. One of these situations includes infectious organisms such as *Mycobacterium* tuberculosis, protozoa, fungi, specific viruses (like HIV, Hepatitis C, Lymphocytic choriomeningitis virus), and other parasites, which have the ability to resist host defenses and can linger in the tissue for extended periods (75, 77). In some viruses, like SARS-CoV-2, even after SARS-CoV-2 mRNA and proteins cannot be detected, COVID-19 sequelae related to multi-organ chronic inflammation persist for months (78). It has also been shown that the intestinal microbiota can stimulate the immune system and, in the case of excessive stimuli, affect the formation of inflammation (79, 80). In these instances, the inflammatory response might not resolve appropriately or may be improperly regulated, leading to a state of chronic inflammation.

### 3.2 Chronic inflammation in cancer

In the context of cancer, chronic inflammation has been linked to various steps involved in tumorigenesis, including cellular transformation, promotion, survival, proliferation, invasion, angiogenesis, and metastasis. One of the first connections between chronic inflammation and cancer was stablished back in the nineteenth century by the German pathologist Rudolf Virchow, who identified chronic irritation, manifested as a chronic inflammation, as a promoter of cancer (8). In the last decade, our comprehension of the inflammatory microenvironment within cancerous tissues has reinforced Virchow’s hypothesis, and the connections between cancer and inflammation are beginning to hold significance in terms of prevention and treatment (81). The interplay between the two is complex and multifaceted. On one hand, it has been found that in some types of cancer inflammatory conditions are present before a malignant change occurs; and, on the other hand, oncogenic changes can induce an inflammatory microenvironment that promotes the development of tumors (9). Inflammation facilitates tumor progression and treatment resistance, whereas the induction of acute inflammation can promote anti-tumor immunity (82).

The factors that can cause chronic inflammation and raise the risk or progression of cancer include infections (such as *Helicobacter pylori* for gastric cancer and mucosal lymphoma; papillomavirus and hepatitis viruses for cervical and liver carcinomas, respectively), autoimmune diseases (like inflammatory bowel disease for colon cancer), and inflammatory conditions with unclear origins (such as prostatitis for prostate cancer) (83, 84). Cancer-related inflammation, which is considered the seventh hallmark of cancer, is also closely connected to genetic instability (9).

The molecular mechanisms underlying the relationship between cancer and chronic inflammation are complex and multifaceted. One significant process involves the production of reactive oxygen and nitrogen species (RONS) by immune cells present at the inflammation site (85). These molecules, while integral to normal immune responses, can induce DNA damage in excess or when chronically produced. This genotoxic stress could potentially lead to the accumulation of genetic mutations, which is a fundamental prerequisite for the oncogenic transformation of cells. Simultaneously, inflammatory cytokines like tumor necrosis factor-α (TNF-α), interleukins (IL-1, IL-6), and others, released during chronic inflammation, stimulate cell proliferation and inhibit apoptosis (86, 87). This abnormal cell survival and propagation set the stage for the expansion of mutated cell populations. Further, inflammatory mediators, such as vascular endothelial growth factor (VEGF), foster angiogenesis, facilitating the establishment and nourishment of growing tumors (88). This process allows tumors to receive the necessary nutrients and oxygen while disposing of waste products, effectively supporting their continued growth and survival. The inflammatory microenvironment also influences the immune response in a way that paradoxically fosters cancer development. Typically, immune cells like cytotoxic T cells and NK cells can recognize and eliminate cancerous cells. However, in a chronically inflamed context, factors like Transforming Growth Factor-β (TGF-β) can suppress these cells’ function and promote an immunosuppressive microenvironment (89). Concurrently, tumor-associated macrophages (TAMs) and myeloid-derived suppressor cells (MDSCs), which are often abundant in chronically inflamed tissues, can promote cancer progression by secreting pro-tumorigenic factors and inhibiting effective anti-cancer immunity (90).

Therefore, the relationship between chronic inflammation and cancer is underpinned by a complex interplay of molecular factors and cellular behaviors. Understanding these dynamics is crucial for developing effective strategies for cancer prevention and therapy. In general, it has been found that deletion or inhibition of inflammatory mediators inhibits the development of experimental cancers (91). For instance, long term use of non-steroidal anti-inflammatory drugs (NSAIDs) reduce the mortality caused by cancers such as colon and breast cancer (92, 93). However, it should be taken into account that these substances can damage the balance of beneficial bacteria in the gut and cause inflammation of the intestinal walls (known as leaky gut), which can lead to the release of toxins and inflammation throughout the body (75).

### 3.3 Chronic inflammation in aging

Examining the third vertex of this conceptual triangle, the intimate relationship between aging and chronic inflammation is captured in the term ‘*inflammaging*’, a fusion of ‘inflammation’ and ‘aging’ (94, 95). This term signifies a low-grade, persistent inflammation that emerges with advanced age and is implicated in numerous age-related diseases, such as atherosclerosis, arthritis, diabetes, and dementia. Thus, aging plays a role in chronic inflammation as our immune system deteriorates with time, making it less effective in controlling inflammation. This can cause dysfunction in organs, cells, and tissues (96), making us more susceptible to diseases and ultimately, death (97) (immunosenescence). These facts seem to indicate a direct correlation between genes that regulate the immune response and longevity (96, 97). In addition, there are certain lifestyle factors that can increase the risk of chronic inflammation, such as smoking, excessive alcohol consumption, stress and sedentarism. These risk factors are more common with age (98). Aging results from the accumulation over time of detrimental changes at the molecular and cellular levels, and ultimately at the level of tissues and organs, resulting in disease and increased risk of morbidity and mortality (99). These are known as primary hallmarks, which would include genomic instability, telomere attrition, epigenetic alterations, loss of proteostasis and disabled macroautophagy. The antagonistic hallmarks are our body’s responses to mitigate the damage produced by the primary hallmarks and include cellular senescence, mitochondrial dysfunction and deregulated nutrient-sensing (51). The integrative hallmarks appear when the damage caused by the primary and antagonistic hallmarks becomes too great for the tissue’s homeostatic mechanisms to repair or compensate for: stem cell exhaustion, altered intercellular communication, dysbiosis and chronic inflammation (6, 51). However, the relationship between chronic inflammation and the other hallmarks is not only one of cause and effect, but also of effects. Such inflammatory process consequences may reduce the ability of the organism to fight against new infections, thus affecting the way the immune system responds to pathogens. For example, chronic inflammation can cause the immune system to over-respond to an infection, which can damage body tissues, increase the risk of complications and also favor cell proliferation (100). In addition, chronic inflammation can affect how the immune system recovers from infections, which can prolong recovery time and increase the risk of reinfection. This can help to understand why individuals who have chronic inflammation are more susceptible to SARS-CoV-2 (101, 102).

Molecularly, the aging process is associated with an increase in the systemic levels of pro-inflammatory cytokines, such as TNF-α and IL-6 (95). Some genetic variations can impact a person’s risk for these diseases by affecting the production of these cytokines. On the other hand, individuals who have a genetic predisposition to produce low levels of proinflammatory cytokines or high levels of anti-inflammatory cytokines may have a higher probability of reaching advanced age (74, 103) Therefore, proinflammatory molecules present in circulation can accurately predict age-related illness and death (104, 105).

## 4 The CAIn Triangle: Lipid composition alterations as an indispensable cornerstone

In addition to the well-acknowledged association of persistent inflammation, the CAIn Triangle’s three interconnected points—cancer, infection, and aging—also share an often-undervalued commonality: the disturbance of lipid balance in the cellular membranes of affected cells.

Lipid membranes are highly complex and dynamic systems formed by different types of lipids combined in specific ratios that depend on several factors including diet, age, and environment exposure (106). The variation in headgroups and aliphatic chains allows the existence of >1,000 different lipid species in any eukaryotic cell (107). With the rapid advancements in lipidomics, distinct lipid profiles have been identified in cancerous cells, cells infected with bacteria or viruses, and aging or senescent cells, emphasizing the need for more intensive research (108). Therefore, changes in the lipid constituents of the cells involved in these conditions are suggested as valuable indicators for surveillance, offering prospective uses in the domains of health promotion, disease detection, and therapeutic intervention.

**Figure 4:**
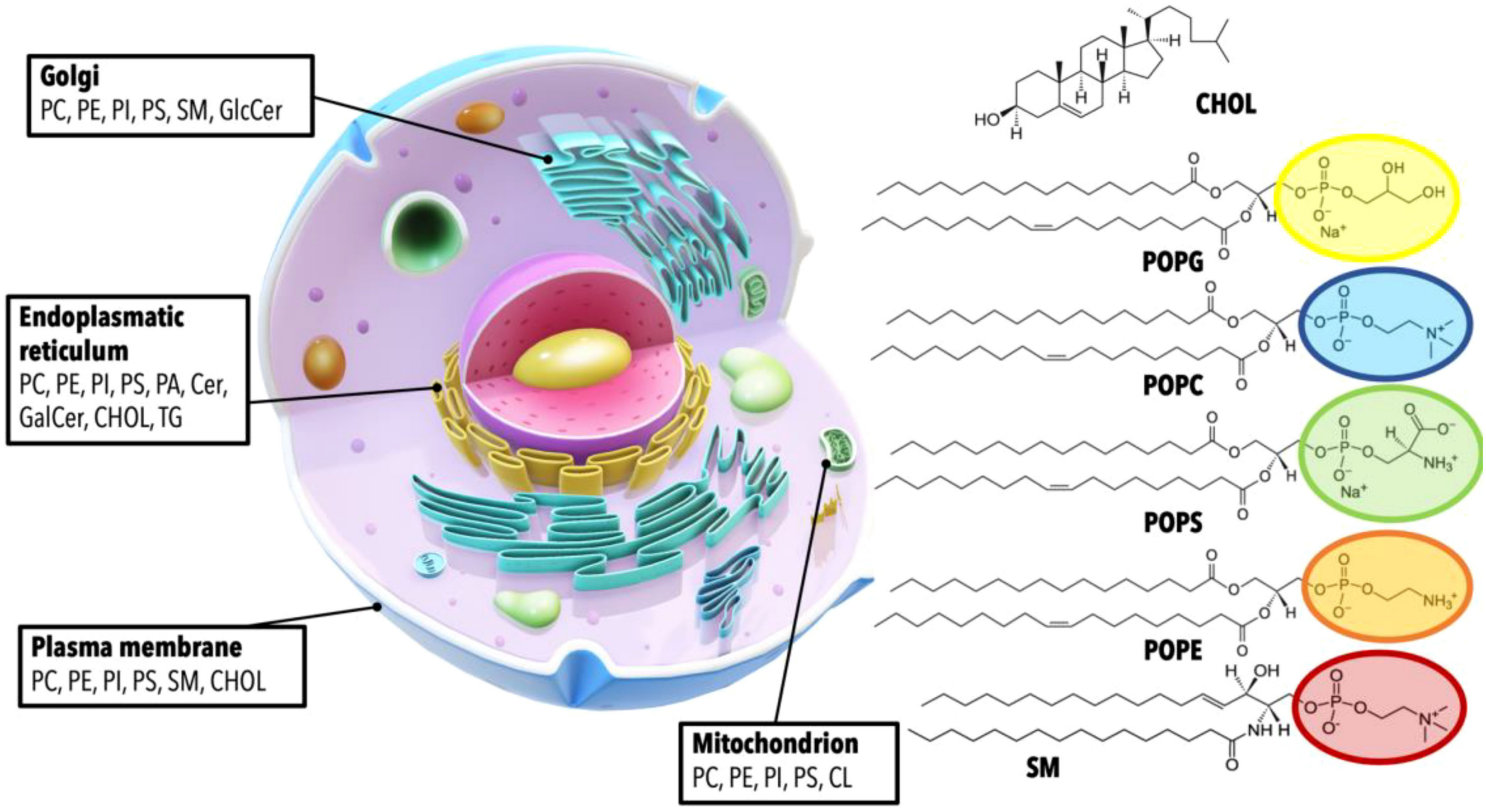
Cellular organelles and their characteristic lipid compositions. This figure provides a detailed visualization of the cellular landscape, highlighting the distribution of various lipids within different cellular compartments. Each organelle is annotated with its respective complement of lipids, demonstrating the unique lipid makeup that characterizes the Golgi apparatus, endoplasmic reticulum, plasma membrane, and mitochondrion. The specific lipids depicted include Phosphatidylcholine (PC), Phosphatidylethanolamine (PE), Phosphatidylinositol (PI), Phosphatidylserine (PS), Sphingomyelin (SM), Cholesterol (CHOL), Glucosylceramide (GlcCer), Ceramide (Cer), Galactosylceramide (GalCer), and Triglyceride (TG), with their respective chemical structures presented. A generalized structure of glycerophospholipids is shown with the acyl chains represented by 1-palmitoyl-2-oleoyl (PO) groups, which serve as an example of the fatty acid composition of these lipids.

Lipids constitute approximately 50% of the mass of most cell membranes, although this proportion varies depending on the type of membrane (**Figure 4**). Located on the outermost layer, the plasma membrane primarily consists of three lipid categories: glycerophospholipids, sphingolipids, and sterols, asymmetrically distributed within the bilayer’s two leaflets. The most abundant phospholipid is phosphatidylcholine (PC), constituting approximately 45-55% of the membrane’s phospholipid content (109). The second most abundant is phosphatidylethanolamine (PE), which accounts for 15-25% of the membrane’s phospholipid content. Phosphatidylserine (PS), with an estimated 10-15% prevalence, is typically found in the intracellular layer of the membrane. Other glycerophospholipids include phosphatidylinositol (PI), phosphatidylglycerol (PG), cardiolipin (CL) and phosphatidic acid (PA). The most common sphingolipids are ceramides (Cer), sphingomyelin (SM), and gangliosides (GM). Finally, cholesterol (CHOL) is the major non-polar lipid found in mammalian cell membranes. The chemical heterogeneity of lipids in cell membranes is immensely vast and differs substantially not only between specific organisms but also within different membrane types in the same cell (110, 111). The concentration of sphingolipids and cholesterol, for example, relative to glycerophospholipids, varies across different cellular structures: it is highest in the plasma membrane, intermediate in the Golgi apparatus, and lowest in the endoplasmic reticulum. The intricate relationships between these constituent lipids in cellular membranes play a pivotal role in numerous pathologies. Imbalances or dysregulations in these lipids can trigger a host of diseases, including but not limited to, metabolic disorders, neurodegenerative diseases, and cancer. In fact, dysregulation of the plasma lipidome is characteristic of chronic inflammatory diseases (112, 113). It is essential to emphasize that these lipid imbalances or dysregulations, along with chronic inflammation, form an intersecting point within the CAIN Triangle. Hence, a comprehensive understanding of lipid distribution and regulation in cellular membranes could unlock valuable insights for the prevention, diagnosis, and treatment of cancer, infection and aging.

### 4.1 Lipid alterations in cancer

When a cell transforms into a cancerous state, significant alterations occur in its lipid composition. This lipidomic remodeling is one of the hallmarks of cancer, enabling the malignant cell to sustain rapid growth, resist apoptosis, and adapt to its microenvironment. Changes in the levels of different types of lipid molecules in cell membranes have been reported in various types of cancer (114, 115). The asymmetrical distribution of lipids across the two leaflets of a normal eukaryotic cell membrane, with PS (which under physiological or logical conditions of pH is negatively charged) and PE typically located in the inner leaflet, and PC and SM in the outer leaflet, is actively maintained by ATP-dependent enzymes, flippases and floppases. This arrangement is disturbed in cancerous conditions, with PS and PE becoming exposed on the outer leaflet, altering the structural and biophysical properties of cancer cell membranes and potentially affecting drug penetration and efficacy (116). Traditionally, the exposure of PS is recognized as a hallmark of apoptotic cells, serving as an ‘eat me’ signal to PS receptors on immune cells, thereby instigating cellular clearance by macrophages (117). Apart from its role in apoptosis, PS is also implicated in modulating immune responses. Specifically, PS exposure on cancer cells is known to foster immunosuppression within the tumor microenvironment. This exposure amplifies the activity of natural killer cells and dendritic cells while steering the polarization of tumor-associated macrophages (TAMs) towards an anti-inflammatory (M2) phenotype (31). Currently, several agents have been developed to target PS, and some of them are being investigated in clinical trials as combination therapies for different types of cancer. However, conflicting outcomes have emerged both in laboratory studies and clinical trials, leading to uncertainty regarding the effectiveness of PS-targeting agents (118). On the other hand, CHOL, which typically decreases membrane fluidity under normal conditions, plays a crucial role in modulating the resistance to chemotherapy and the metastatic properties of cancer cells. When cancer cells prepare for metastasis, they have a lower concentration of cholesterol, which maximizes fluidity, among other factors (114). Conversely, higher membrane cholesterol levels account for chemotherapy resistance.

Lipidomics, specifically MS-based shotgun lipidomics strategies, have been widely used in cancer research (12). They help identify changes in lipid metabolism, including alterations in lipid abundance, redistribution, and phospholipid composition. These changes could be potential biomarkers for cancer diagnosis, prognosis, and treatment. For instance, in prostate cancer, cholesteryl ester species (20:1, 24:5, 24:4, 18:1, and 22:6), Cer species (d18:2-20:0 and d18:1-20:1), NEFA species (22:3) and TAG species (58:1) have shown a significant increase in cancerous tissues compared to non-cancerous ones (119). The remarkable upregulation of these lipid species, particularly cholesteryl ester, indicates potential biomarkers for prostate cancer diagnosis and progression. In breast cancer, lipid species profiling has proven useful for diagnosis. Distinct patterns of lipid species, including PI (16:0-16:1) and PI (18:0-20:4), were identified as potential biomarkers (120). In addition, combinations of LysoPC (LPC), PC, and cholesterol esters have shown high diagnostic accuracy in distinguishing early-stage breast cancer from benign breast disease (121). In ovarian cancer, higher LPC and lower PC and TAG with specific fatty acid chains were found in patients’ plasma (122). A combination of LPC, PC, and TAG species is proposed as potential biomarkers for distinguishing ovarian cancer from healthy individuals. Lipidomics studies in laryngeal cancer patients showed that certain lipid species were significantly altered (123). Specifically, the levels of Cer, CerG1, SM, PC, PC-O, PE, PI, PS, and ChE were increased, while LPC, LPC-O, LPE, LPE-p, and DG levels were decreased. Notably, lysophospholipids with palmitoyl or arachidonic acid acyl chains showed a remarkable decrease, whereas phospholipids with palmitoyl or arachidonic acid acyl chains exhibited an increase. In colorectal cancer, specific lipid profiles undergo significant alterations compared to matched normal tissue (124). Tumor samples exhibit higher levels of mono- and polyunsaturated LPC species, increased proportions of SM species with 32-34 carbons, and elevated sphingosine-based Cer with longer chains. Conversely, shorter Cer chains and TG species with <53 carbons are reduced, while polyunsaturated TG species with 56 carbons are enhanced in tumours. On the other hand, nine lipids (LPC 16:0, 18:0, and 20:4; phosphatidylcholines 16:0–18:1, 16:0–18:2, 18:0–18:1, 18:0–18:2, and 16:0–22:6; and triglycerides 16:0–18:1–18:1) were identified as the features most important for early-stage lung cancer detection (125).

### 4.2 Lipid alterations in infection

In various infections, including bacterial, viral, tuberculosis, and parasitic, alterations occur in plasma lipid levels. Total cholesterol, LDL-C, and HDL-C levels usually decrease, while plasma triglyceride levels tend to increase or remain normal despite malnutrition (126). There’s also a reduction in apolipoproteins A-I, A-II, and B levels. Intriguingly, the quantity of small, dense LDL rises when overall LDL-C levels fall.

During viral infections such as those caused by HIV, HCV, HBV and even SARS-CoV-2, there’s a systemic metabolic shift from anabolism to catabolism leading to a chronic form of the disease (127, 128). This shift brings about long-term health consequences, one of the most notable being dyslipidemia, a condition characterized by abnormal lipid levels in the bloodstream. Lipidomic research has revealed specific alterations in lipid profiles in HIV patients, including changes in seven lipid classes and 83 individual lipid species (129). Particularly, diacylglycerols (DAGs), triglycerides (TAGs), and various other lipids like PI and PG are associated with increased cardiovascular disease (CVD) risk. HIV can affect fatty acid synthesis, which could lead to further lipid profile modifications. These lipid changes may vary based on the phase of HIV replication and antiretroviral therapy status. Some studies have aimed to elucidate the link between lipid alterations and chronic HCV infection. Individuals suffering from chronic HCV infection display a unique lipid profile in contrast to healthy individuals. Importantly, a significant difference in several lipids has been noticed between mild and severe intrahepatic inflammation grades (IGs), indicating their potential use as innovative noninvasive indicators of intrahepatic IG (130). HBV virus infection, on the other hand, is associated with a global alteration of serum lipidomics in non-alcoholic fatty liver disease patients (131). Upregulation of PC, choline plasmalogen (PC-Os) and downregulation of free fatty acids, LPCs, dominated the HBV-related lipidomic characteristics. Another example is the role played by the lipid profile of COVID-19 patients both during the acute infectious process, the evolution of the disease and even in the medium-long term sequelae. Interestingly, changes in lipid levels had been observed in patients who had recovered from the SARS outbreak in 2003. Wu et al. conducted a long-term study, tracking 25 SARS survivors for 12 years post-infection. Their findings indicated elevated levels of phosphatidylinositol and lysophosphatidylinositol (132). In the context of SARS-CoV-2, lipid metabolism disruptions have been observed across all stages of the disease, including recovery (13, 133–135). Y. López-Hernández showed early-stage alterations in acylcarnitines and glycerophospholipids (PCs and LPCs) in patients admitted to emergency rooms (136). Chen and colleagues have reported that even in patients with negative nucleic acid tests (still hospitalized), lipid metabolism remains dysregulated (137). Approximately two months post-discharge, Acosta-Ampudia et al. found that recovered patients continued to exhibit metabolic differences compared to pre-pandemic controls, including altered levels of unsaturated fatty acids like arachidonic and linoleic acid (138). Li et al. associated lipid metabolic disturbances with long-term chronic discomfort and immune dysregulation in COVID-19 survivors six months post-discharge (139). Notably, they reported dysregulated levels of various lipids, including TG, LTB4, PGE2, and several polyunsaturated fatty acids. Even two years post SARS-CoV-2 infection, certain metabolic pathways remain altered (140). Interestingly, within the same lipid family, some lipid species demonstrated decreased levels in post-COVID patients, while others showed an increase. These ongoing irregularities in lipid metabolism may provide insight into the lingering symptoms experienced by patients, particularly those associated with musculoskeletal conditions.

### 4.3 Lipid alterations in aging

The relationship between aging and changes in lipid composition of cell membranes has been extensively studied in both animal and human models, using chromatographic and mass spectrometric techniques (141). A comprehensive longitudinal study in 2020 identified that 40% of age-correlated metabolites were lipids (142). Specific lipid profiles have been associated with human and animal longevity. For example, higher sphingolipid levels have been detected in the blood plasma of centenarians (143). Odd chain fatty acids and ether lipids have been observed to increase in longer-lived animals compared to those with shorter lifespans (144). Another study measured 128 lipid species and identified 19 that were associated with longevity. It was found that in women, there was an increase in sphingomyelins and phosphocholines, while there was a decrease in triglycerides and ethanolamines (145). Jove at al. proposed ceramides as biomarkers of extreme longevity (146), whereas the decrease of PC and PE with age has also been observed in model organisms and could potentially serve as biomarkers for aging and other diseases (147). In 2019, Wong et al. conducted a study that found differences in lipid composition not only as a function of age, but also by sex. Specifically, they observed that women had a higher concentration of total cholesterol, as well as sphingomyelin and phospholipids containing docosahexaenoic acid (148). Recent research has shown that a decrease in LPCs is associated with a decline in the oxidative capacity of mitochondria. Furthermore, decreased levels of this specific lipid (LPC 18:2) have been identified as a predictor of memory decline in older adults (149, 150) and have also been linked to the development of cancer.

Lipid levels in the brain tend to decline after the age of 50, and this decrease is associated with a decline in myelin concentration and a decrease in the percentage of dry matter (151). The first study to observe a direct relationship between aging and lipid alteration was conducted by Burger and Rouser, who found that brain aging also modifies the lipid ratio, resulting in a loss of about 10% of PC, PI, and PE from age 40 to 100 (152). On the other hand, in diseases such as Alzheimer’s (AD) (153), there are alterations in the levels of certain lipids. For example, studies have found a decrease in the concentration of PC (153) and PE (154) and some sphingomyelins (SM18/14:0, SM18/16:1, SM18/17:0) together with an increase in other SM species (SM18/18:1, SM18/18:0) (155) and Cer (156). Additionally, there are changes in cholesterol levels, including a decrease in 24s-hydroxycholesterol and an increase in 27-hydroxycholesterol (157). These changes in lipid composition have been observed in the brain and may play a role in the development and progression of AD.

Recently, the shifts seen in lipid composition, incorporated into a lipidomic clock, have been explored for their capacity to estimate the biological age of organisms such as *C. elegans* (158).

## 5 Conclusions and perspectives

In this study, we have delved into the interconnections between cancer, aging, and infection, resulting in the establishment of the CAIn Triangle as a crucial conceptual framework. While chronic inflammation undoubtedly stands out as a prominent link within the CAIn Triangle, we have shed light on another critical yet often neglected common denominator: the lipidic alteration of cellular membranes in the involved cells. The remarkable progress in the field of lipidomics has uncovered distinctive lipid profile characteristics in cancer, bacterial and viral infections, as well as senescent cells. This breakthrough offers a remarkable opportunity to leverage lipid composition as a precisely targeted therapeutic and diagnostic tool. The ability to analyze lipidic signatures provides not only prospects for prevention and early detection but also facilitates the development of personalized treatments tailored to the individual patients’ lipidic profiles.

### 5.1 Lipid biomarkers for the prevention and diagnosis of CAIn pathologies

Lipids have long been utilized in disease diagnosis and evaluating overall health. For instance, cholesterol and triglycerides have served as established indicators of heart health and dietary habits for several decades (159). However, the field of lipidomics has expanded significantly in recent years, enhancing our collective knowledge of the roles lipids play as biomarkers in various diseases associated with the CAIn Triangle (160–164).

While the fields of genomics and proteomics have well-established routine methodologies for measurement and data reporting, the field of lipidomics is still relatively young and faces certain limitations that need to be addressed. The complexity lies in the vast number of unique lipid structures, as exemplified by the impressive collection of 47,189 structures compiled by the Lipid Maps® consortium (107). To accelerate the translation of potential biomarkers, it is essential to develop better methodologies, technologies, and internal standards. These advancements are not only necessary for improving accuracy and precision in readouts across laboratories but also for achieving throughput, cost-effectiveness, and ease-of-use, thereby facilitating the translation of potential biomarkers in lipidomics research. The ongoing lipidomics standardization initiatives and inter-lab engagements are pivotal for attaining the required study quality and lipid biomarker expertise (165–167). These collaborative efforts aim to ensure accurate lipid quantification that can be reliably applied across platforms and laboratories, ultimately enabling the translation of lipid markers into routine clinical use. Moreover, the development of robust quantification methods for lipid panels, regardless of their size, is crucial to meet clinical requirements. Additionally, as similar markers are often linked to different conditions, the utilization of composite biomarkers is likely to provide more comprehensive insights than single lipid biomarkers. The future holds the promise of larger lipid panels, comprising over 100 lipids, to enhance risk assessment and improve disease diagnosis for pathologies connected through the CAIn Triangle. Future technologies should permit the measurement of broader biomarker panels, extending beyond lipids alone, to facilitate accurate disease diagnosis and monitoring at the individual level.

To expedite the adoption of lipidomic profiles in clinical practice, advancements in methodologies and technologies must be complemented by user-friendly interfaces. The utilization of specialized electronic devices, such as lab-on-a-chip technology (168, 169), holds significant promise in this regard. Integrating lab-on-a-chip technology into wearable devices can revolutionize health monitoring, allowing for the continuous or periodic tracking of lipid profiles and enabling the prevention and early diagnosis of severe health conditions. While commercial wearables currently track parameters like heart rate, glucose levels, and blood gas concentrations, they have the potential to expand their capabilities to include lipid-level monitoring. This integration of lab-on-a-chip technology with wearable devices has the potential to greatly enhance our ability to monitor and prevent serious health conditions. With the rapid growth of knowledge in lipidomics, it is only a matter of time before new lipid-based clinical tests and precision health solutions become commonplace, revolutionizing the field of healthcare.

### 5.2 Redefining therapeutic approaches: lipid-targeted interventions in the CAIn Triangle

**Figure 5:**
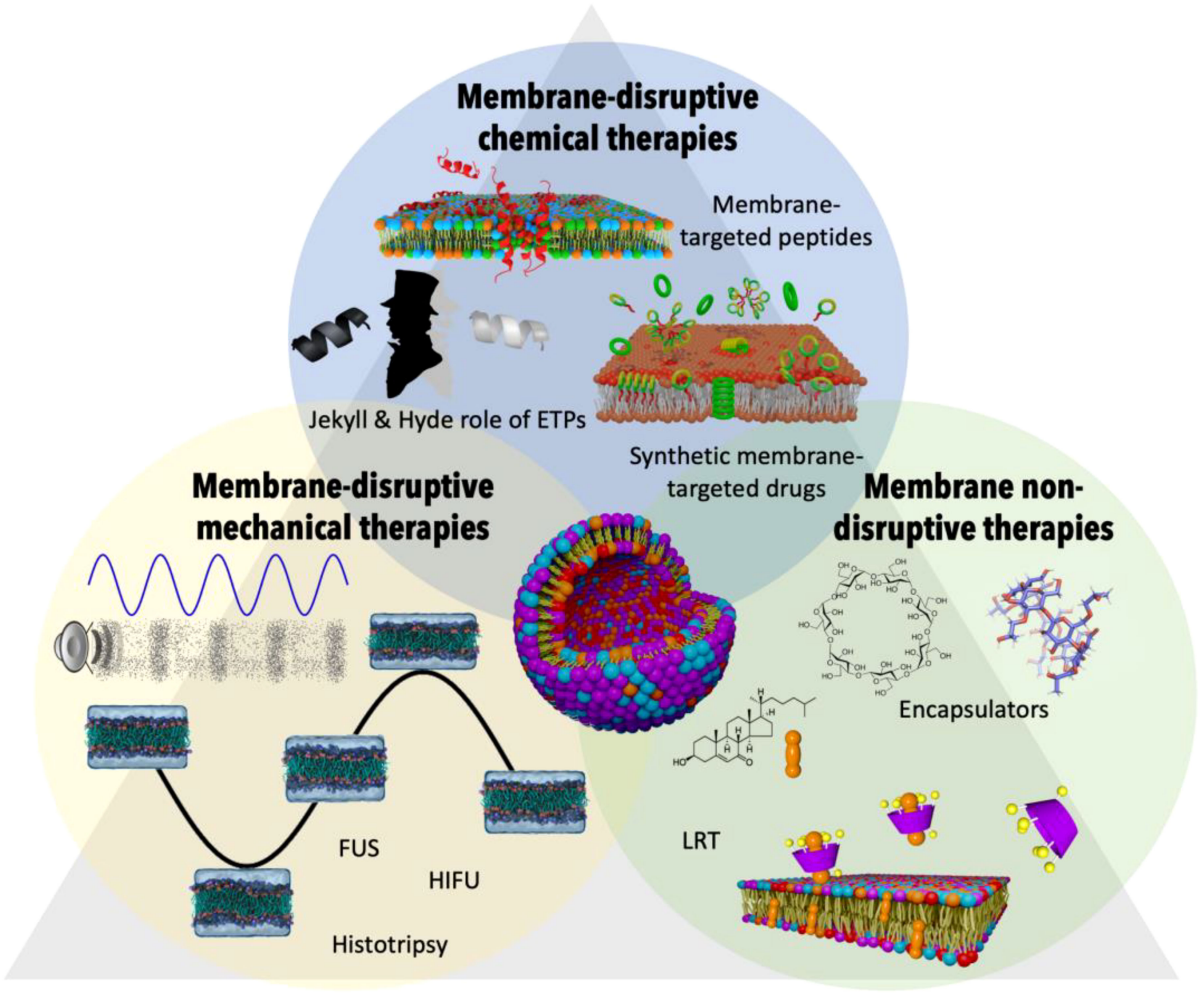
Therapeutic strategies targeting altered membrane lipids in the CAIn triangle. This figure offers a visual summary of various therapeutic interventions aimed at addressing the altered membrane compositions seen in conditions of cancer, infection, and aging. Membrane-disruptive chemical therapies: This group includes membrane-targeted peptides and synthetic membrane-targeted drugs that interact with and disrupt the membrane lipids of cancerous or otherwise diseased cells. The concept of the ‘Jekyll & Hyde role of ETPs’ is highlighted, noting the potential for both therapeutic and harmful effects of these peptides. Membrane-disruptive mechanical therapies: Depicted are techniques such as Focused Ultrasounds (FUS), High-Intensity Focused Ultrasounds (HIFU), and Histotripsy, which physically disrupt membrane integrity to target diseased cells, leveraging the mechanical forces to induce therapeutic effects. Membrane non-disruptive therapies: This section portrays encapsulator molecules that actively remove harmful lipids such as oxidized cholesterol from the membrane without disrupting its structure. Alongside, Lipid Replacement Therapy (LRT) is a complementary approach that seeks to restore healthy lipid composition, thereby aiding in the maintenance of membrane integrity and overall cellular function within the context of CAIn diseases.

The interconnection of lipid alterations among the vertices of the CAIn Triangle presents a remarkable opportunity to redefine treatment approaches by targeting cellular membranes. By identifying, characterizing, and leveraging the physical-chemical and mechanical vulnerabilities of the cells involved in the CAIn Triangle, we can develop interventions that specifically address altered membranes, mitigate their effects, and eliminate toxic compounds (**Figure 5**).

One promising avenue is the development of drugs that target the altered lipid compositions in cell membranes, enabling the treatment of affected cells while preserving the integrity of healthy cells. Traditionally, most drugs have relied on direct protein interactions to exert their effects. However, as our understanding of membrane lipids grows, we recognize their pivotal role in regulating cellular functions and the activity of membrane proteins, offering an alternative molecular target (170–172).

Nature has ingeniously exploited the specific lipid composition of pathological cells, tissues, and even pathogenic agents like viruses, bacteria, and fungi to develop defense mechanisms. Endogenous Therapeutic Peptides (ETPs), small cationic peptides within the innate immune system, play a crucial role in this process (173). Membrane-targeted ETPs recognize abnormal lipid patterns in cell membranes, disrupting lipid bilayers and leading to the destruction of corresponding cells (174). This mechanism operates independently of protein receptors and occurs whenever the lipid composition deviates from that of healthy cell membranes, regardless of the underlying cause, such as infection, cancer, or aging. Building upon these insights, one approach for developing new treatments involves creating molecules that specifically target and destroy cells with abnormal lipid profiles, similar to the action of ETPs (170, 173, 175, 176). Interestingly, an inflammation process can be triggered by the activity of ETPs in a pathological environment. Thus, despite their protective power, ETPs might also function as a ‘double-edged sword’ by injuring not only the pathogen but also the host, by triggering additional chronic inflammation. Exacerbated autoimmune responses in infections, including COVID-19, have been attributed to the direct interaction of pathological membrane compositions with ETPs (13). Overexpression of ETPs also contributes to aging through cytotoxic effects (177). Intriguing yet contradictory findings have demonstrated that while some ETPs have anti-tumoral activity and are under-expressed in solid tumours, others are overexpressed and pro-tumorigenic (178). This dual nature of ETPs paves the way to new treatment strategies in diseases affected by chronic inflammation based on the use of anti-ETPs to cancel out their undesired effects (179). Following the same principle acting in anti-ETPs therapies, it should be possible to remove the excess of harmful ETPs in particular situations using more attractive pathologic lipid compositions (therapeutic vesicles) as antagonists of their effect.

On the other hand, pathological cell membranes can be selectively targeted and disrupted through non-chemical alternative methods, offering potential therapeutic interventions. The mechanical and physicochemical properties of biological membranes heavily depend on their lipid composition (180, 181). The use of mechanical perturbations to selectively target and disrupt pathological cell membranes opens up exciting prospects for therapeutic interventions. One particularly promising approach is the utilization of focused ultrasonic wave pulses with specific amplitude and frequency to eliminate pathogenic cells while leaving healthy cells intact. This targeted intervention using focused ultrasound offers significant advantages over chemical alternatives. By precisely delivering ultrasonic waves to specific areas, it minimizes the impact on healthy tissues, resulting in fewer side effects. Unlike systemic drug treatments that may affect the entire body, this approach allows for localized and controlled disruption of pathological membranes. Advancements in Focused ultrasound (FUS) techniques (182), such as High-Intensity Focused Ultrasounds (HIFU) (183–185), Low-intensity pulsed ultrasound (LIPUS) (182), and histotripsy (186), continue to refine the precision and effectiveness of this therapeutic strategy. By utilizing specific frequencies, amplitudes, and periodic pulses, these interventions can induce mechanical disruption in targeted tissues. Ongoing research aims to optimize the parameters of amplitude and frequency to ensure maximum disruption of pathogenic membranes while minimizing impact on surrounding healthy cells. Moreover, the development of advanced imaging techniques, such as real-time ultrasound imaging, enables accurate visualization and guidance during the intervention. This facilitates the precise targeting of pathological areas and provides valuable feedback on the treatment’s progress and efficacy. While challenges remain in fully understanding the complex relationship between membrane composition and mechanical properties, ongoing investigations and systematic studies are essential to drive progress in this field. The establishment of comprehensive databases that capture relevant information will aid in the development of innovative therapies that leverage the mechanical vulnerabilities of altered cell membranes. Advancements in ultrasound technology and imaging techniques, coupled with continued research, will pave the way for transformative interventions that improve patient outcomes and contribute to the advancement of personalized medicine.

Besides selectively destroying membranes with abnormal lipid composition through chemical or mechanical techniques, it is equally important to develop methods that repair or alleviate the effects of these alterations, aiming to restore cellular function. Dietary modulation or supplementation represents a simple strategy that can modify the lipid composition and counteract pathological changes (187–190). Lipid Replacement Therapy (LRT) (191) is one such approach that involves the administration of oral supplements containing phospholipids and antioxidants to replace pathogenic lipids that accumulate in various clinical pathogenic conditions. LRT supplements containing GPL (glycerophospholipids), antioxidants, and other ingredients have shown promise in repairing and replacing oxidatively-damaged membrane GPL, thereby restoring the activities and functions of cellular membranes, organelles, cells, tissues, and organs, improving the quality of life in aging and chronic illnesses (189). Clinical trials have demonstrated their effectiveness in reducing symptoms associated with loss of mitochondrial and other cellular functions, as well as improving the quality of life in patients with various chronic illnesses and age-related functional decline (189). The potential of LRT and other interventions aimed at restoring lipid composition is vast. Continued research and development in the field of LRT, including investigations into optimal dosages, long-term effects, and combination therapies, will further advance this therapeutic approach. Advances in lipidomics techniques, coupled with personalized medicine approaches, may pave the way for individualized lipid-based therapies based on patients’ specific lipid profiles, enabling more tailored and effective treatments.

Furthermore, advancements in the field of molecular encapsulators offer a more complex alternative, enabling the restoration of pathological lipid compositions to their healthy state (192). These encapsulators can remove harmful substances, allowing the membrane to regain its integrity and functionality. Ongoing research aims to optimize the encapsulation process, enhance the selectivity of encapsulators, and explore their effectiveness in various pathological conditions.

As a final noteworthy observation, it is important to highlight that lipid formulations have demonstrated the ability to influence the immune system through their physicochemical properties, including size, lipid composition, PEPgylation, and surface characteristics (193, 194). This intriguing finding opens up new possibilities in immunotherapy. By designing vesicles with compositions similar to those of pathogenic or pathological cells, it may be possible to stimulate the immune system in a targeted manner. This innovative approach has the potential to facilitate the development of vaccines specifically aimed at preventing inflammation and related immune conditions. Exploring the immunomodulatory properties of lipid-based formulations offers exciting prospects for the future of vaccine design and the advancement of personalized immunotherapeutic interventions.

## Author contributions

DC-T: Data curation, Investigation, Writing – original draft, Writing – review & editing. AB-G: Data curation, Investigation, Writing – original draft, Writing – review & editing. AS-G: Data curation, Investigation, Writing – original draft, Writing – review & editing. FS-L: Data curation, Investigation, Writing – original draft, Writing – review & editing. AC: Data curation, Investigation, Writing – original draft, Writing – review & editing. PA-R: Data curation, Investigation, Writing – original draft, Writing – review & editing. ÁP: Data curation, Investigation, Writing – original draft, Writing – review & editing, Conceptualization, Funding acquisition, Methodology, Supervision. RG-F: Conceptualization, Data curation, Funding acquisition, Investigation, Methodology, Supervision, Writing – original draft, Writing – review & editing.
